# The value of perforator flap reconstruction in painful soft tissue calcifications

**DOI:** 10.1007/s10238-024-01421-0

**Published:** 2024-08-13

**Authors:** Loraine Kouba, Adriano Fabi, Kathrin Glatz, Anna Thoma, Ana Lariu, Maximilian Burger, Thierry Schweizer, Dirk J. Schaefer, Elisabeth A. Kappos

**Affiliations:** 1 Department of Plastic, Reconstructive, Aesthetic and Hand Surgery, University Hospital Basel, Spitalstrasse 21, 4031 Reconstructive Basel, Switzerland; 2 Faculty of Medicine, University of Zurich, Zurich, Switzerland; 3 Faculty of Medicine, University of Basel, Basel, Switzerland; 4 Department of Pathology, University Hospital Basel, Basel, Switzerland; 5 Department of Rheumatology and Pain Medicine, Bethesda Hospital Basel, Basel, Switzerland; 6 Iuliu Hațieganu University of Medicine and Pharmacy, Cluj-Napoca-Napoca, Romania

**Keywords:** Soft tissue calcification, Dystrophic calcification, Calcinosis, Pedicled flap reconstruction, Reconstruction, Quality of life

## Abstract

Soft tissue calcifications frequently cause debilitating pain and functional impairments, considerably affecting patients’ quality of life. As they are rare entities, evidence remains sparse, especially regarding treatment effectiveness and recurrence rates. While both pharmacological and surgical treatments may alleviate symptoms, complete resection is currently believed to prevent long-term recurrence of deposits. To improve understanding and raise awareness for soft tissue calcifications, the goal of this study was to review the current state of treatment and to compare benefits and possibilities of flap reconstruction versus simple excision in improving quality of life. Furthermore, we include a successful case report of complete resolution of symptoms following quadruple perforator flap reconstruction. By systematic literature review, studies published in MEDLINE between 1980 and 2024 reporting on surgical treatment and outcome of soft tissue calcifications were included, in addition to a detailed description of our case report. A total of 53 studies reporting on 197 patients with soft tissue calcifications were included. Simple surgical excision was the most commonly (85.9%) employed procedure, demonstrating a substantial recurrence rate of 13.3%. In contrast, no patients who underwent radical excision experienced recurrence. Dermal matrix grafts and flap reconstruction were successfully used in patients requiring substantial tissue coverage, highlighting their value in complex defect reconstruction following radical excision. The combination of complete surgical resection and flap reconstruction reduces recurrence rates and improves postoperative outcomes and quality of life of these patients, supporting early radical surgical intervention as the gold standard treatment for soft tissue calcifications.

## Introduction

Soft tissue calcifications (STCs) are defined as depositions of insoluble calcium salts in soft tissues, which can be asymptomatic or lead to debilitating pain with respective loss of function [1]. The etiologies of soft tissue calcifications are diverse and can be categorized in five subgroups: dystrophic, metastatic/metabolic, idiopathic, iatrogenic and calciphylaxis [2]. Dystrophic calcifications are the predominant type of soft tissue calcifications, typically appearing within pre-damaged tissues and presenting normal serum calcium and phosphorus levels [2]. They are frequently associated with connective tissue diseases, such as systemic sclerosis, dermatomyositis, and systemic lupus erythematosus [3–8]. Other common etiologies include cutaneous neoplasms, panniculitis, infections and trauma e.g. burns or repetitive subcutaneous injections. Inherited disorders are considered rare [9, 10].

Initial management typically encompasses non-surgical approaches, such as physical therapy and analgesic, as well as anti-inflammatory therapy [11, 12]. Pharmacological treatment approaches remain controversial, as no medical treatment has consistently proven reliable [13–15]. Surgical intervention may be considered when conservative measures fail to provide adequate relief. However, the extent of surgical intervention, e.g. narrow-margin excision versus radical resection, remains understudied and unexplored. To support future decision making, this systematic review aimed to compare long-term recurrence rates and quality of life in patients undergoing surgical treatment for soft tissue calcifications.

### Case Report

A 65-year-old female patient was referred to our outpatient clinic for treatment of diffuse and painful soft tissue calcifications. Her medical history revealed chronic use of subcutaneous pethidine injections administered over a four-year period to manage persistent pain following various orthopedic knee revision surgeries associated with a total knee prosthesis for osteoarthritis. Despite maintaining skin integrity, the patient reported a progressive hardening around the injection sites after two years, evolving into debilitating, extremely painful calcifications affecting the proximal areas of all four extremities, impairing her mobility and daily activities. Apart from the tender skin lesions, the patient was in good health with no features of an autoimmune disorder.

**Fig. 1:**
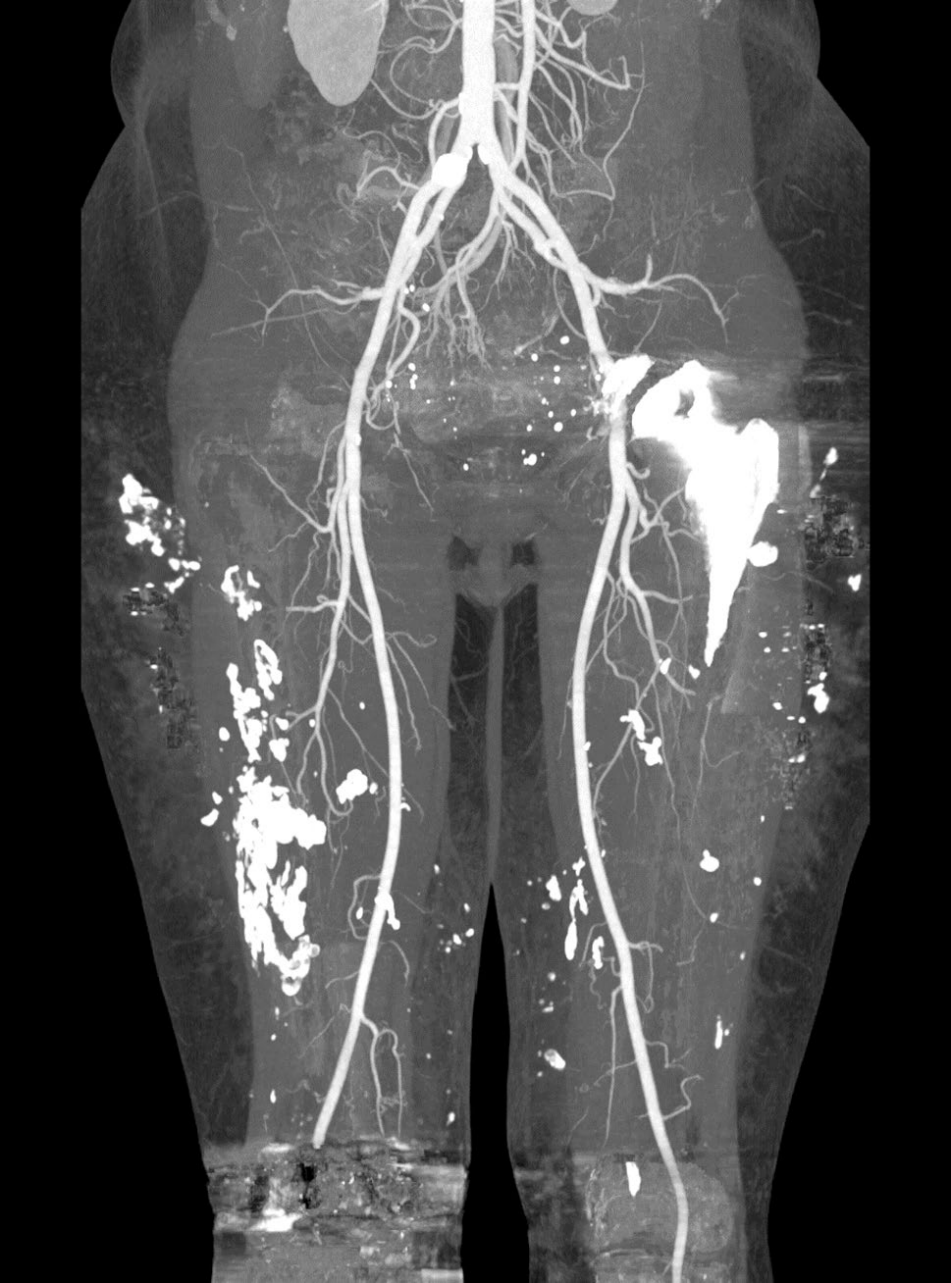
Preoperative CT angiography demonstrating soft tissue calcifications in both thighs

**Fig. 2:**
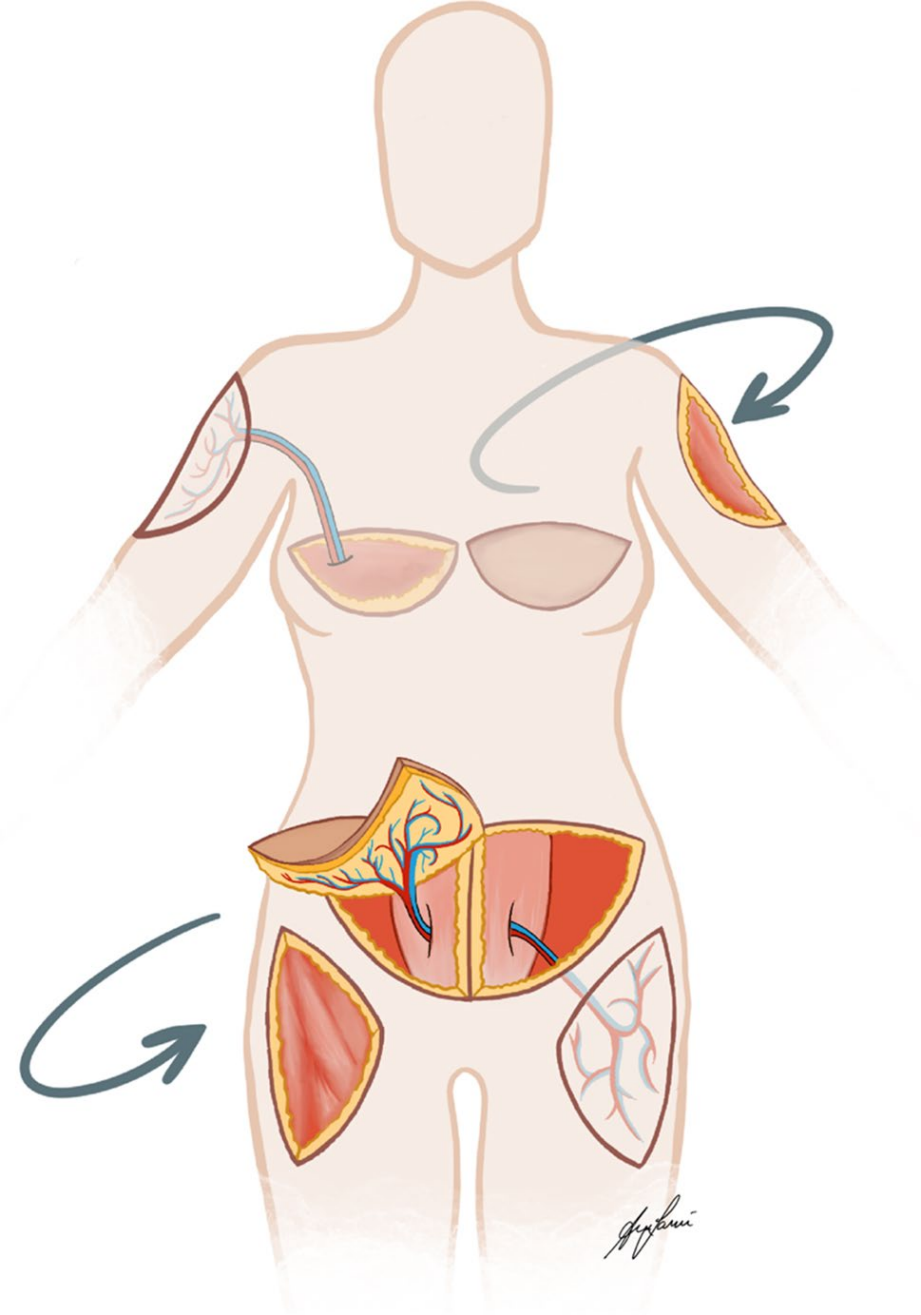
Schematic illustration of all performed surgical interventions

**Fig. 3:**
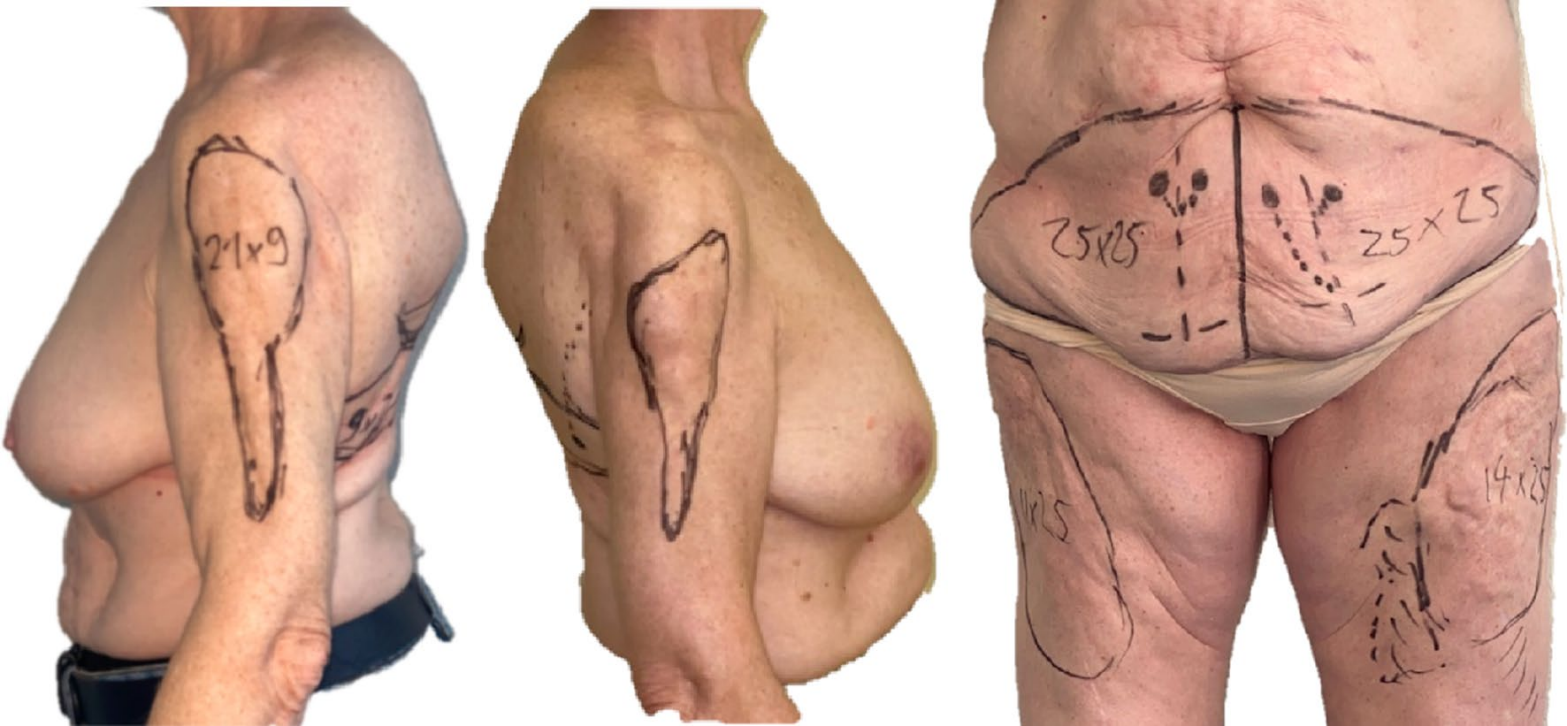
Preoperative markings of resection areas in accordance to the painful calcifications

Physical examination revealed large, firm subcutaneous masses infiltrating the overlying skin on both arms (8 × 10 cm) and thighs (15 × 20 cm). Diagnostic imaging, including preoperative CT angiography and SPECT/CT, revealed focal calcifications in the subcutaneous fatty tissue of the bilateral thighs and panniculitis (Fig. 1). Blood values, including calcium, phosphorus, parathormone and creatinine, were within normal range. Hence, dystrophic iatrogenic calcifications of the soft tissues attributed to chronic subcutaneous pethidine injections was determined the underlying diagnosis. However, the multidisciplinary non-surgical treatment approach involving pain management, physical therapy, orthopedics, and rheumatologists achieved very limited relief only. Consequently, a shared decision was made to proceed with extensive surgical resection of the painful lesions followed by reconstructive surgery with quadruple pedicled perforator flaps across three separate operations (Figs. 2 and 3).

**Fig. 4:**
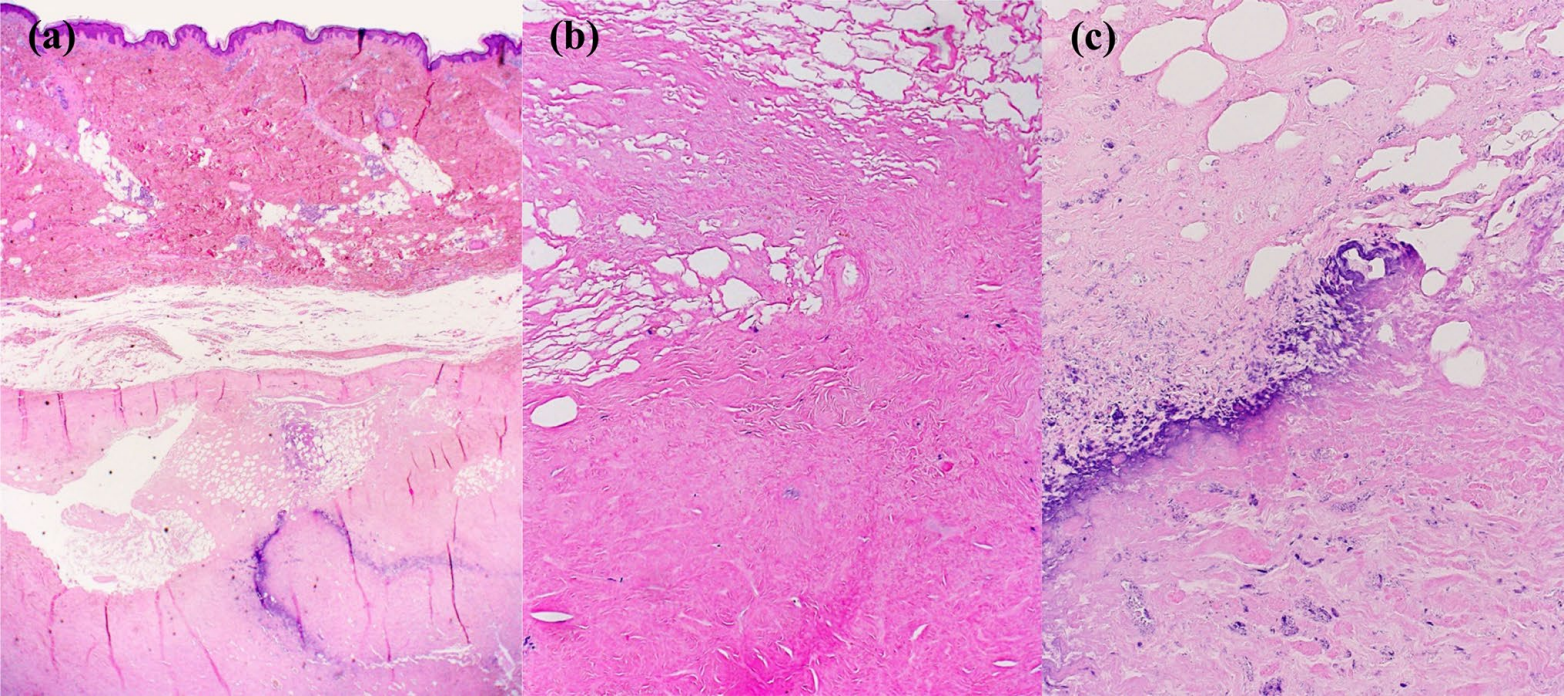
Microscopic examinations revealed **a** old scarred fatty tissue necrosis and cell-poor sclerosis with dystrophic blue-violet calcifications. (H&E, 20x); **b** old scarred fatty tissue necrosis with shadowy adipocytes and cell-poor sclerosis (H&E, 100x); **c** old scarred fatty tissue necrosis with shadowy recognizable adipocytes and cell-poor sclerosis with dystrophic blue-violet calcifications (H&E, 200x)

The primary surgery included an extensive resection of calcifications and radial nerve neurolysis in the area of the upper arm, utilizing an ipsilateral single thoracodorsal artery perforator (TDAP) flap for soft tissue coverage. Histopathological analysis of the excised tissue demonstrated extensive replacement of subcutaneous fat by scar tissue. Only small areas of shadowy scarred necrotic fatty tissue were recognizable within the sclerotic areas. The adjacent fascial tissue was superficially scarred and had a thickness of up to 15 mm. The sclerosed soft tissue showed focal blue-violet calcium deposits (dystrophic calcification of necrotic or degenerative tissue), while the dermis remained unaffected. No ossification could be observed and, therefore, there was no evidence of myositis ossificans. There were also no inflammatory changes, as seen in scleroderma, and no sclerosis of the dermis accompanied by inflammatory changes, as seen in morphea. These scleroderma-like changes in the deep cutaneous soft tissue were most consistent with old, scarred tissue necroses with dystrophic calcifications induced by several years of subcutaneous injections of pethidine. The cell-poor hyalinized areas were suggestive of amyloid. However, Congo red staining was negative in the fibrosed tissue. The final pathological diagnosis confirmed diffuse dystrophic calcifications (Fig. 4).

**Fig. 5:**
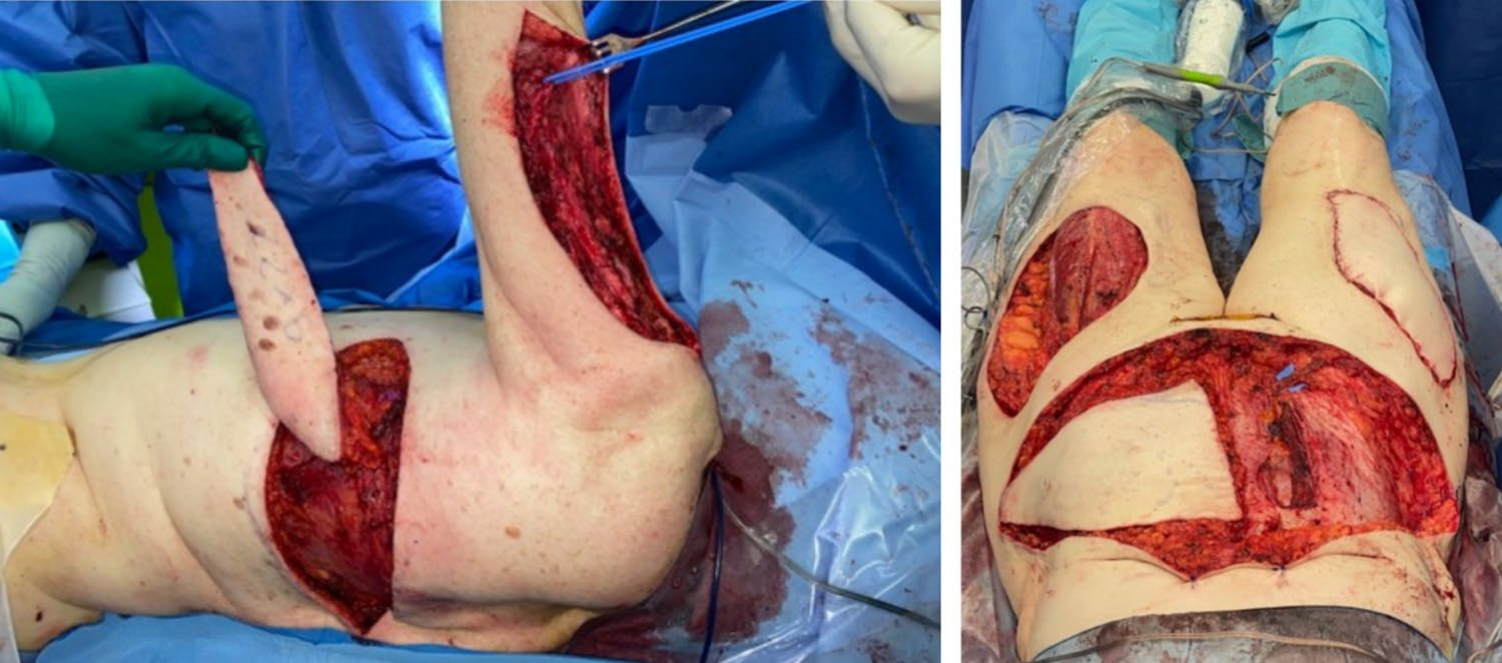
Intraoperative TDAP-flap elevation and resection area on the upper arm (left image) and intraoperative image of DIEP-flap reconstruction of the resection areas of the thighs (right image)

**Fig. 6:**
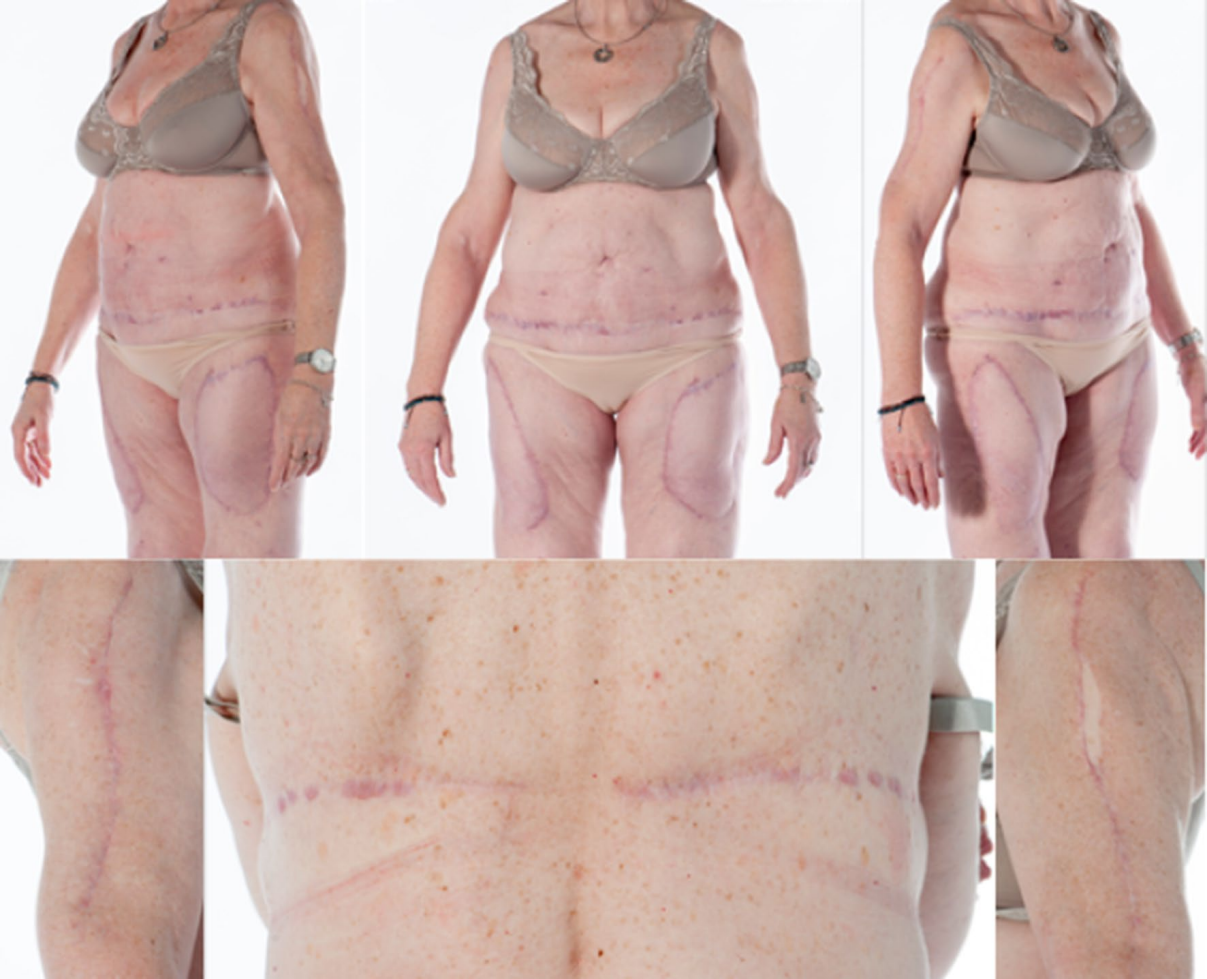
Postoperative documentation at the one-year follow-up. Complete survival of all flaps (top pictures) with well-healed incision wounds (bottom pictures) in a completely pain-free patient

Following the primary surgery, the patient experienced complete pain relief. This significant improvement in her condition led to the decision to proceed with further surgical treatment. The second surgery six months later included wide resections of the soft tissue calcification of the left arm, again with the need for neurolysis of the left radial nerve on the level where tissue resection was performed. A second ipsilateral single perforator TDAP flap was used for reconstruction **(**Fig. 5**)**. Again, the patient reported complete resolution of all symptoms. After another three months, the patient underwent final resection of the painful, calcified soft tissue areas on the lateral proximal thighs bilaterally, with reconstruction using pedicled double-perforator deep inferior epigastric perforator (DIEP) flaps. Intraoperative bilateral venous congestion was resolved by venous supercharging. A postoperative hematoma of the lower abdomen and both thighs necessitated surgical evacuation. Subsequently, the patient experienced a smooth recovery with an otherwise uneventful postoperative course. Complete pain relief and drastic amelioration in quality of life was reported after the final surgery. Surgical treatment was finalized with volume reduction and further de-epithelialization of both TDAP flaps after six months. At the one-year follow-up, the patient showed no sign of pain recurrence and expressed substantial satisfaction with the result (Fig. 6).

## Methods

A systematic literature review was conducted in accordance with the PRISMA 2020 checklist following criteria based on the PICO schema to analyze any differences between pharmacological treatment versus narrow margin surgical excision versus radical debridement and flap reconstruction in terms of recurrence rates and quality of life in patients with soft tissue calcifications [16].

### Search strategy

The MEDLINE/PubMed database was searched for articles published between 1980 and 2024 with no language restrictions using the following free text sequence: “Surgical treatment of soft tissue calcifications” as of February 2024. Literature search was performed by the shared first authors. All titles and abstracts collected from the initial search were systematically scanned for relevance. The full texts of all relevant articles were then obtained and reviewed for suitability. Of all articles meeting inclusion criteria, the reference lists were secondarily screened for additional eligible studies.

### Study selection

Papers screened for eligibility included case reports, case series, systematic reviews, letters to the editor and randomized controlled trials published in all languages that incorporated the surgical treatment and outcomes of any soft tissue calcifications. Studies were included if they reported 1) soft tissue calcifications; 2) a minimum of one case of surgical excision with or without adjunct pharmacological treatment and 3) a documented post-operative outcome. Exclusion criteria were 1) review articles such as literature reviews or commentaries; 2) diagnosis of calcification due to malignancy or bone calcification disorders; 3) non-surgical treatment approach only and 4) articles with no reported postoperative patient outcome. Given the rarity of soft tissue calcifications, single case reports were not excluded.

### Data extraction and definition

Manual data extraction included the following: author, country, publication date, study design, methodological data, number and age of included patients, type and extent of soft tissue calcification, choice of intervention, reconstructive soft tissue coverage, outcome and author’s conclusions. If not further mentioned, surgery was generally viewed as a narrow-margin excision. If any of the words “extensive”/”aggressive”/”radical” or similar were explicitly used to describe the intervention, or if healthy tissue was debrided in addition to the calcified deposits, the surgical intervention was classified as an extensive debridement.

### Statistical analysis

Descriptive statistics was used to report data.

## Results

### Search results

**Fig. 7:**
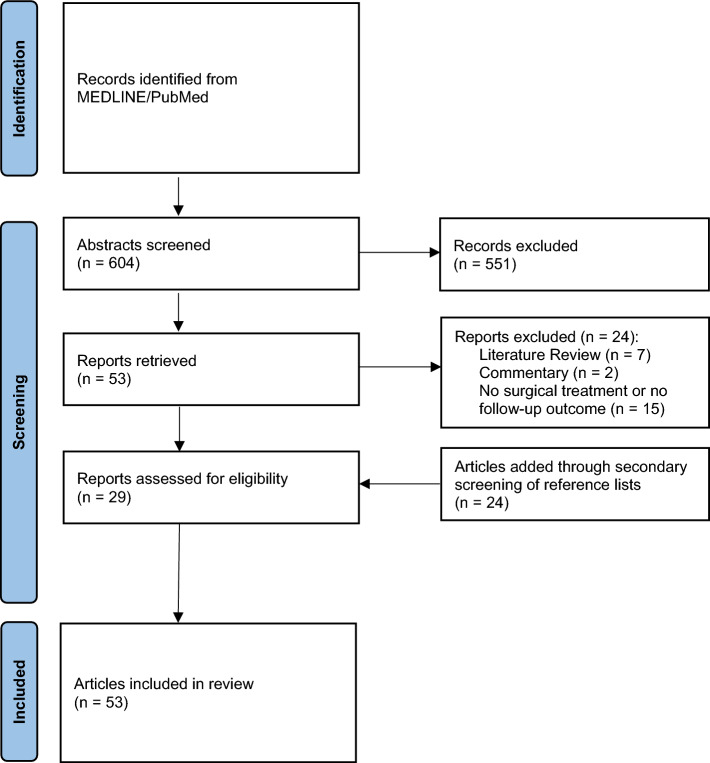
Flow diagram of search strategy for paper selection

Of the 604 abstracts assessed during the preliminary assessment, 53 articles were identified for potential eligibility (Fig. 7). After full-text evaluations, 24 studies were excluded due to their review nature, non-surgical treatment approach and/or lack of follow-up, leaving 29 articles for inclusion. Additionally, 24 studies were manually added through secondary search of all reference lists. Hence, 53 articles were included in this systematic review, comprising 1 original article, 3 case series and 49 case reports.

### Study characteristics

The sample sizes spanned from 1 to 111 cases, for a total of 197 patients aged between 6 months [17] and 84 years [18]. The predominant diagnoses included tumoral calcinosis (n = 163, 82.7%), cystic myonecrosis (n = 12, 6.1%) and calcinosis cutis (n = 12, 6.1%).

### Pharmacological management of soft tissue calcifications according to our literature review

Pharmacological treatment of soft tissue calcinosis largely depends on the underlying cause, yet remains highly controversial, as no medical treatment has consistently proven reliable. In hopes of reducing recurrence rates, pharmacological treatment was utilized in 20 (10.2%) patients included in this systematic literature review. The most common therapy (n = 12, 60%) involved the administration of phosphate binders, such as aluminum hydroxide or calcium acetate [13, 19–23]. Furthermore, dietary modifications with phosphorus, calcium and/or potassium-restriction were recommended for 7 (35%) patients [13, 21–23]. Other medication included warfarin [24], local steroid/anesthetic injections [12], low-dose minocycline [25], calcium channel blockers [24], acetazolamide [22] and bisphosphonates [26].

### Pharmacological management of soft tissue calcifications according to further literature evaluation

In the literature, isolated cases have achieved regression and long-term remission with a phosphorus depletion diet combined with the use of phosphate chelators, such as aluminum hydroxide or calcium acetate, in patients with tumoral calcinosis or calcinosis cutis [27–29]. Nonetheless, others experienced no therapeutic effect when using aluminum hydroxide for prevention of recurrence [19, 23, 30]. Cukierman et al. documented complete resolutions of calcinotic lesions in two out of three patients undergoing low-dose warfarin treatment [31]. These findings were reflected by Yoshida and Torikai, who achieved improvement of calcinosis in an ulceration of the finger following a low-dose warfarin regimen [32]. Contrarily, Chamberlain et al. observed no regression with warfarin therapy [24]. Alabaz et al. advocated for bisphosphonates, as no new lesions occurred during therapy and accelerated progression occurred after cessation. However, they failed to demonstrate regression of calcifications during the proposed therapy [26]. Local steroid/anesthetic injections have proven inefficient and have not achieved long-term symptomatic relief [5, 12]. Hence, pharmacological management has not been accepted as a standard treatment to reduce calcinosis or prevent recurrence due to the heterogenicity of clinical responses.

### Surgical excision

For patients with localized, superficial calcifications, non-invasive modalities such as extracorporeal lithotripsy and laser surgery have achieved notable reductions in size and even long-term remissions in patients with calcinosis cutis [24, 25]. Despite their effectiveness, these interventions may require multiple treatment sessions and may not adequately address more extensive calcifications. In contrast, large and extensive nodules typically require surgical excision given their tendency for postoperative complications, functional impairments, and infiltration of underlying joints [12, 33, 34].

In this systematic literature review, surgical intervention was the treatment of choice for 192 (97.5%) patients, with the majority (n = 165, 85.9%) undergoing straightforward excision of calcified deposits with immediate tissue closure. Radical tissue debridement was performed in 13 (6.8%) patients. Less invasive procedures including partial removal or simple incision and drainage were less common (n = 4, 2.1%), as were parathyroidectomy (n = 4, 2.1%) or renal transplantation (n = 4, 2.1%). Interestingly, 17 (32.1%) of the 53 articles explicitly recommend surgical excision as the treatment modality of choice.

### Recurrence rates and treatment effectiveness

Postoperative recurrence of soft tissue calcification was observed in 26 (13.2%) patients, occurring between one month [35, 36] and two years [25] postoperatively. Specifically, 22 of the 165 (13.3%) patients undergoing straightforward surgical excision experienced recurrence. Notably, patients receiving radical and aggressive debridement showed no recurrence, compared to three of the four (75%) patients undergoing partial removal of deposits only, hence underscoring the potential efficacy of comprehensive surgery over partial removals only. For patients undergoing dialysis or in cases of severe hyperparathyroidism, parathyroidectomy may achieve long-term remission [20, 37]. Of the four patients undergoing parathyroidectomy, full resistance to treatment was observed in two cases [13, 20]. However, if parathyroidectomy achieves incomplete improvement, renal transplantation may be a viable last resort option, which achieved full remission in both remaining cases [20]. The postoperative recurrence/remission state was not clearly reported for 12 (6.1%) patients [4, 14, 37–45].

### Possibilities for soft tissue coverage

Soft tissue reconstruction was necessary in 10 (4.8%) cases. Four surgical teams (Johnson et al.[46], Al-Sukhni et al.[3], Lee et al.[42] and Tan et al.[47]) used dermal matrix grafts or split-thickness skin grafts either alone or with an artificial dermis. Similarly, Lykoudis et al. described the use of bilateral V–Y advancement gluteal fasciocutaneous flaps after radical debridement in the lumbosacral area achieving full long-term remission [11]. In a notable case of massive ectopic calcification following a lower extremity fracture, Schmitt et al. demonstrated the effectiveness of a free functional gracilis muscle transfers for covering extensive tissue defects [48]. Similarly, Jassal et al. utilized a contralateral rectus abdominis muscle flap in combination with a split-thickness skin graft for reconstruction of the leg after radical excision of calcified myonecrosis [49]. Aydin et al. used a homologous Achilles tendon graft to reconstruct the distal biceps muscle in two patients where tumoral calcinosis infiltrated the distal biceps tendon [50]. In contrast, Amati et al. utilized a reverse homodigital artery flap from the ulnar side of the index finger for tumoral calcinosis of the hand [51].

### Quality of life

While brief mentions of symptom improvement after surgical excision suggest potential positive impacts on quality of life [51, 52], none of the 53 included papers assessed changes in quality of life. Similarly, comprehensive studies using patient-reported outcome measures (PROMs) were completely absent, indicating a significant gap in the literature and emphasizing the need for inclusion of validated PROMs into clinical practice.

## Discussion

In this systematic literature review, a total of 53 studies comprising of 197 patients reporting on the treatment of soft tissue calcifications were identified. The findings from this review indicate that pharmacological interventions have not been accepted as a standard treatment to reduce calcinosis or prevent recurrence, primarily due to limited effect and substantial heterogeneity in clinical outcomes. While the most promising pharmacological treatment options include low-dose warfarin [31, 32] and phosphate deprivation via low-phosphate diets and phosphate binders [27–29], larger studies are still needed to validate these potential treatment options.

Surgical excision, on the other hand, is a reliable treatment option for soft tissue calcifications. However, current literature underscores a concerning trend towards high recurrence rates, with one notable patient having undergone a total of 36 operations [22]. While partial debulking may provide short-term relief, it is associated with an increased risk of recurrence and infection, particularly if calcium deposits remain exposed [22, 23, 30, 53]. This systematic review found that three of the four (75%) included patients undergoing partial removal experienced recurrence [13, 25, 36]. Hence, current consensus suggests complete surgical removal of calcification as the treatment of choice, especially in patients experiencing pain, deformity, or functional impairment [17, 28].

The application of dermal matrix grafts and split-thickness skin grafts following radical debridement has proven effective for soft tissue coverage following radical debridement [3, 42, 46, 47]. Furthermore, flap reconstruction presents a viable option, which has shown excellent postoperative results, with no patient experiencing postoperative recurrence after extensive debridement of calcifications [11, 48, 49, 51].

In summary, surgical excision has consistently been shown to provide enduring relief from soft tissue calcifications, with aggressive excisions correlating with reduced recurrence risk. While these sophisticated surgeries and reconstructions may initially appear excessive, they should be considered in light of the massive suffering and impairment of quality of life associated with soft tissue calcifications. In addition, current evidence advocates for early surgical intervention to prevent the progression of calcifications into ulcerations or the compression of adjacent structures, highlighting the benefits of a proactive, “complete and early” surgical approach [52, 53]. However, patients frequently undergo protracted journeys of mostly ineffective, off-label medication and physical therapy, often without substantial improvement, before finally receiving surgical treatment. Hence, the findings of this systematic literature review support timely referral to surgical specialties to prevent unnecessary delays and optimize clinical outcomes in this progressive disease, emphasizing the superior long-term benefits of surgical treatment, possibly in combination with pharmacological treatment, over a purely conservative approach.

### Quality of life

Because the surgical treatment of soft tissue calcifications is meant to achieve both, improvement of functionality and avoidance of recurrence, quality of life is an essential factor in evaluating the indication for and effectiveness of surgical modalities. While certain studies mentioned a reduction of pain with subsequent improvement in daily functioning, none of the 53 articles explored quality of life for patients undergoing conservative or surgical interventions for soft tissue calcifications. The absence of studies highlights a significant gap in the current literature, underscoring the necessity for future research to utilize patient reported outcome measures (PROMs) when performing symptom-oriented surgery. We suggest the use of the Patient and Observer Scar Assessment Scale (POSAS) for clinical use in patients with extensive scarring following soft tissue calcifications [54].

## Limitations

Although this is the first systematic literature review on the surgical treatment of soft tissue calcifications, it is important to acknowledge its limitations. While the predominance of case series and case reports among the included studies suggest a low level of evidence, the rarity of soft tissue calcifications underscores the importance of reporting and compiling all cases. Hence, this study provides an extensive overview of current treatment strategies for soft tissue calcifications.

## Conclusion

Surgery is an effective treatment option for soft tissue calcifications, with radical debridement achieving lower recurrence rates than simple excisions. The integration of flap reconstruction and skin grafting following radical debridement emerges as a promising strategy to reduce recurrence for patients with advanced soft tissue calcifications. Despite the technical challenges of using pedicled perforator flap surgery, this study encourages its use in extensive tissue defects. There is a notable lack of literature on the quality of life for patients with soft tissue calcifications.

## Data Availability

No datasets were generated or analysed during the current study.
